# Supplementary material for: Influence of magnetic domain walls on all-optical magnetic toggle switching in a ferrimagnetic GdFe film
Source: Beilstein J Nanotechnol. 2022 Jan 17;13:74–81. doi: 10.3762/bjnano.13.5 (PMC8787351; doi:10.3762/bjnano.13.5)
Supplement: File 1 — Magnetic characterization. [file Beilstein_J_Nanotechnol-13-74-s001.pdf]

BEILSTEIN JOURNAL OF NANOTECHNOLOGY

Supporting Information

for

Influence of magnetic domain walls on all-optical magnetic toggle switching in a ferrimagnetic GdFe film

Rahil Hosseinifar, Evangelos Golias, Ivar Kumberg, Quentin Guillet, Karl Frischmuth, Sangeeta Thakur, Mario Fix, Manfred Albrecht, Florian Kronast and Wolfgang Kuch

Beilstein J. Nanotechnol. **2022,** *13,* 74–81. doi:10.3762/bjnano.13.5

Magnetic characterization

License and Terms: This is a supporting information file under the terms of the Creative Commons Attribution License (https://creativecommons.org/licenses/by/4.0). Please note that the reuse, redistribution and reproduction in particular requires that the author(s) and source are credited and that individual graphics may be subject to special legal provisions.
The license is subject to the *Beilstein Journal of Nanotechnology* terms and conditions: (https://www.beilstein-journals.org/bjnano/terms)

To characterize the magnetic anisotropy, superconducting quantum interference device vibrating sample magnetometry (SQUID-VSM) measurements were performed with a Quantum Design MPMS3 magnetometer on a different sample with identical thicknesses and GdFe composition deposited in the same run. A nominal GdFe thickness of 15 nm was used for the conversion of magnetic moment to film magnetization, neglecting a possible induced magnetic moment of the Pt seed layer.

Magnetization loops at room temperature are shown in Figure S1. The blue loop corresponds to the out-of-plane geometry. It shows a small coercivity on the order of 5 mT, while the red loop measured in the in-plane geometry exhibits a more rounded shape, which does not reach to saturation completely. This shows that the easy axis of magnetization of the sample is out-of-plane at room temperature.

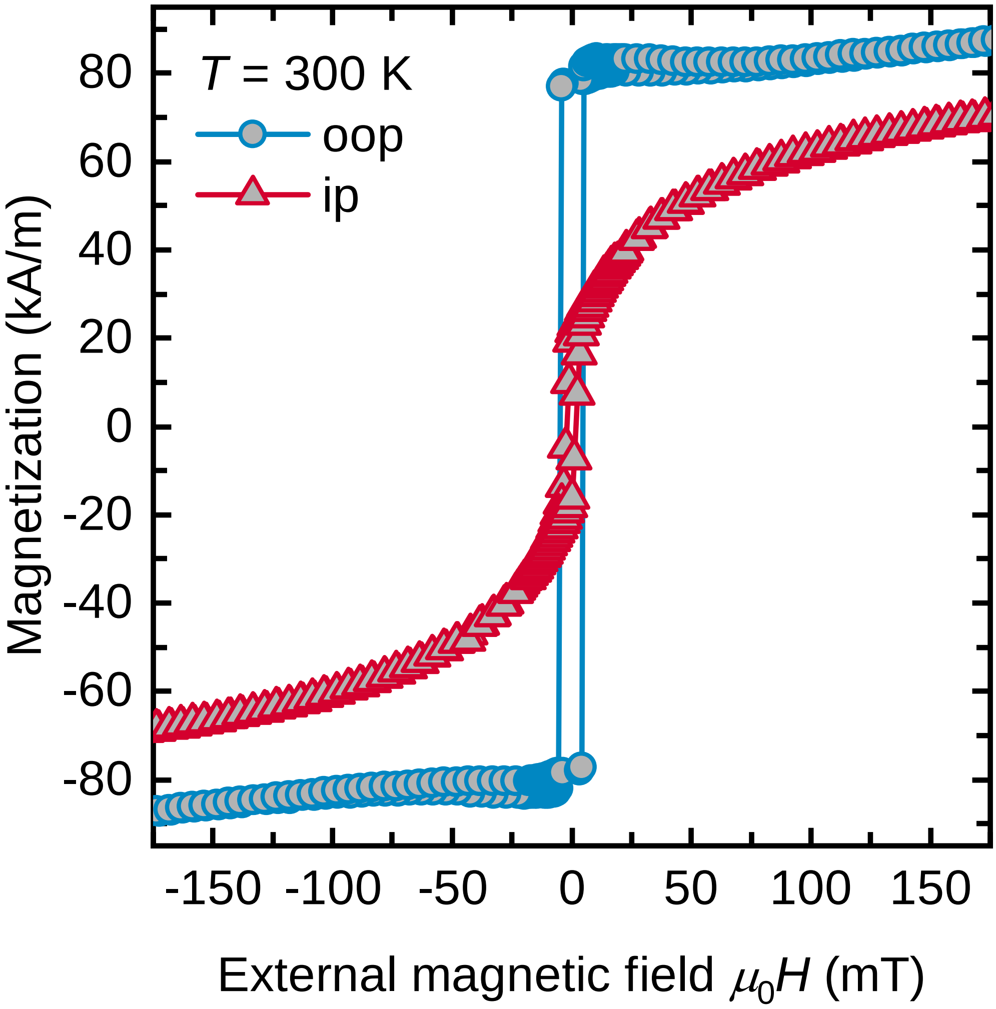

Figure S1: SQUID-VSM measurements of 15 nm $Gd_{26}Fe_{74}$ at a temperature of 300 K in the in-plane (red curve) and out-of-plane geometry (blue curve).

Figure S2 shows SQUID-VSM measurements in the out-of-plane geometry at different temperatures. Raising the temperature from 60 to 120 K increases the coercivity. At 120 K, no hysteresis loop is visible. At 140 K, the coercivity amounts to about 100 mT. By further increasing the temperature, the coercivity decreases until at room temperature, the black loop shows the coercivity of 5 mT. The maximum in the coercivity and a minimum in the remanent magnetization between 100 and 140 K shows that the magnetization compensation temperature of the ferrimagnetic alloy is in that temperature range.

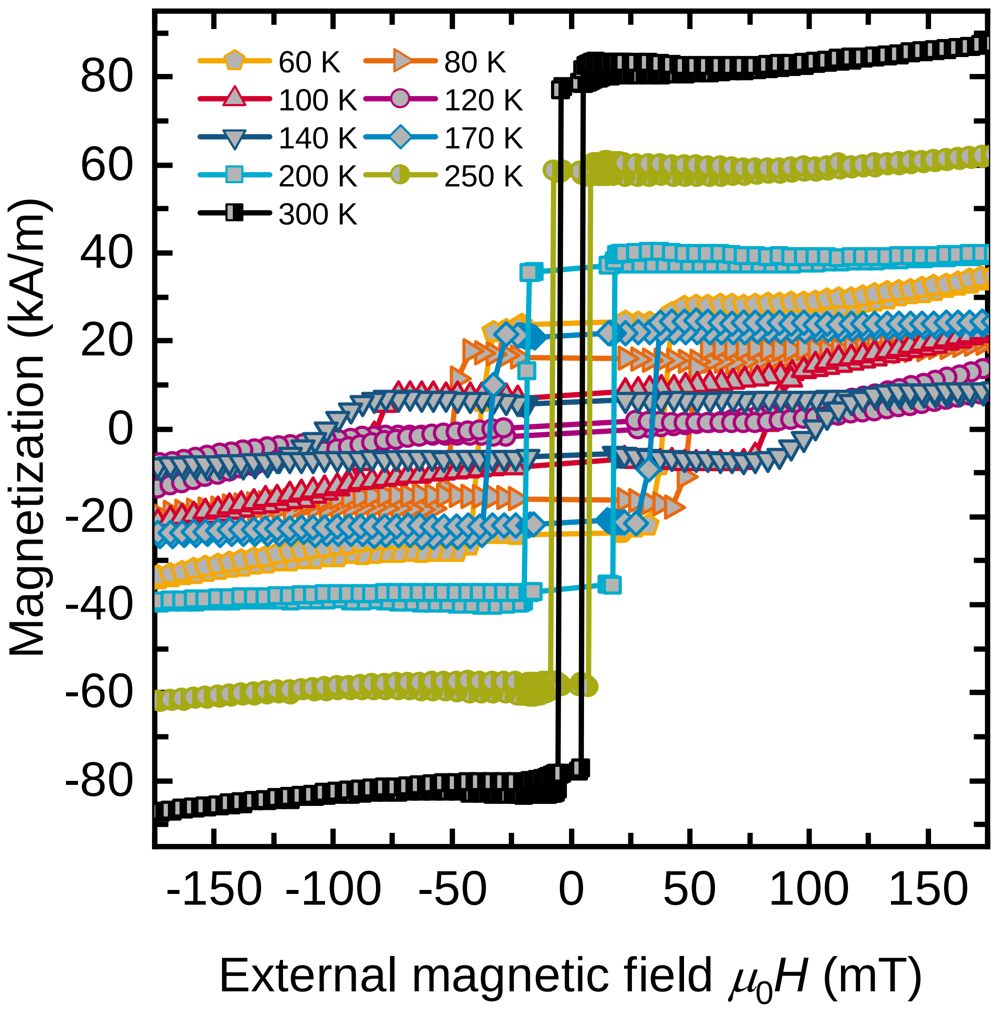

Figure S2: Hysteresis loops of 15 nm $Gd_{26}Fe_{74}$, measured with SQUID-VSM at different temperatures in out-of-plane geometry. The highest coercivity is observed at 140 K, and the lowest one at 300 K. The curve for 120 K does not show a magnetic signal.

Figure S3 shows the remanent magnetization as a function of temperature. It drops to zero at a temperature of 120 K, which means that the magnetic compensation point is at this temperature.

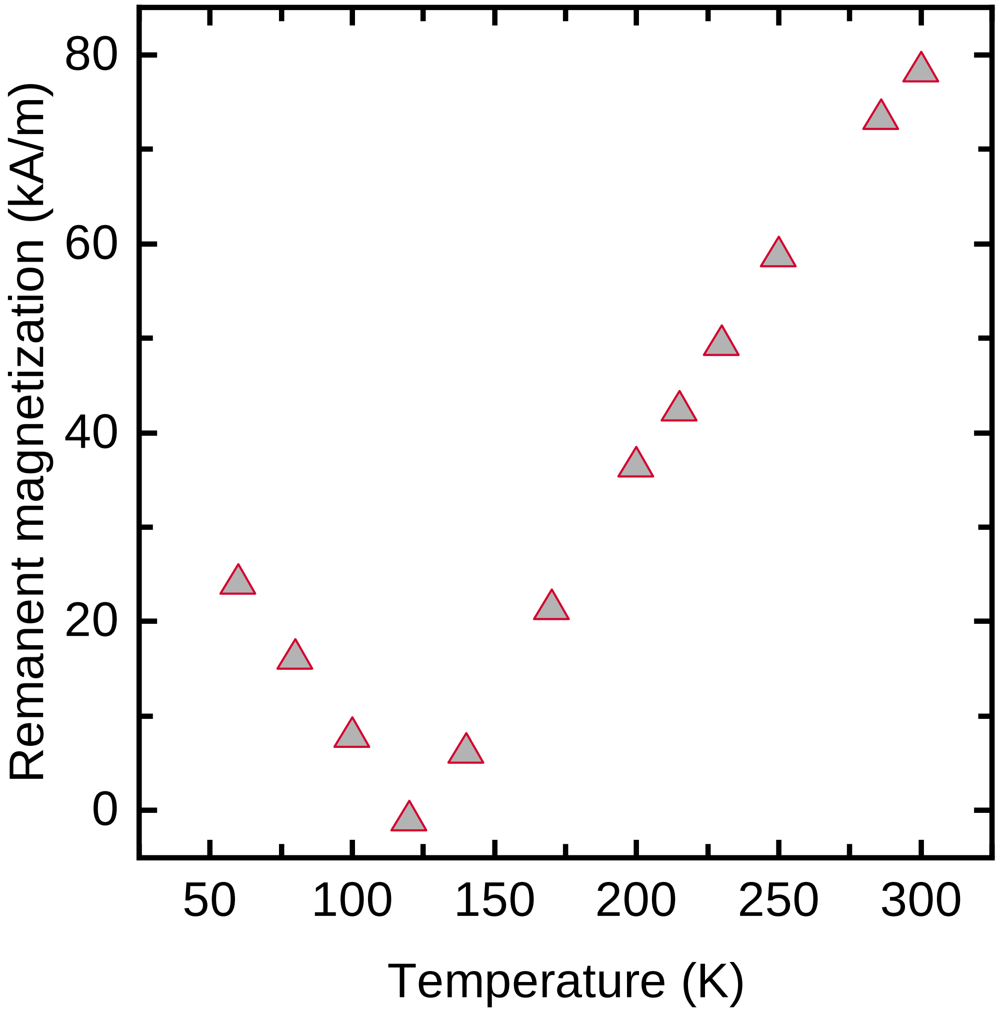

Figure S3: Remanent magnetization from SQUID-VSM measurements of 15 nm $Gd_{26}Fe_{74}$ in the out-of-plane geometry as a function of temperature. The sample shows a magnetic compensation point at around 120 K.
